# HER-2 inhibition in gastric and colorectal cancers: tangible achievements, novel acquisitions and future perspectives

**DOI:** 10.18632/oncotarget.11264

**Published:** 2016-08-12

**Authors:** Valentina Fanotto, Elena Ongaro, Karim Rihawi, Antonio Avallone, Nicola Silvestris, Lorenzo Fornaro, Enrico Vasile, Lorenzo Antonuzzo, Francesco Leone, Gerardo Rosati, Francesco Giuliani, Roberto Bordonaro, Mario Scartozzi, Giovanna De Maglio, Francesca V. Negri, Gianpiero Fasola, Giuseppe Aprile

**Affiliations:** ^1^ Department of Oncology, University and General Hospital, Udine, Italy; ^2^ Department of Oncology, S.Orsola-Malpighi Hospital, Bologna, Italy; ^3^ Gastrointestinal Medical Oncology Unit, National Cancer Institute “Fondazione Giovanni Pascale”-IRCCS, Napoli, Italy; ^4^ Department of Oncology, National CancerInstitute “Giovanni Paolo II”-IRCSS, Bari, Italy; ^5^ Unit of Oncology 2, Azienda Ospedaliero-Universitaria Pisana, Pisa, Italy; ^6^ Department of Oncology, Careggi Hospital, Firenze, Italy; ^7^ Department of Medical Oncology, University of Torino, Candiolo Cancer Institute-FPO-IRCCS, Torino, Italy; ^8^ Medical Oncology Unit, San Carlo Hospital, Potenza, Italy; ^9^ Department of Oncology, ARNAS Garibaldi, Catania, Italy; ^10^ Department of Oncology, University Hospital, Cagliari, Italy; ^11^ Department of Pathology, University and General Hospital, Udine, Italy; ^12^ Medical Oncology Unit, University Hospital, Parma, Italy

**Keywords:** gastric cancer, colorectal cancer, HER2-inhibition, prognosis, predictive factor

## Abstract

HER-2 (ErbB-2, c-erbB2 or Her2/neu), a member of the HER-family, is directly involved in the pathogenesis and progression of several human cancers; as such, it is also often considered as a poor prognostic factor. Following the revolutionary impact of anti-HER-2 therapy in breast cancer patients, the role of HER-2 and its blockade has also been extensively evaluated in other tumor types, including gastric and colorectal adenocarcinoma. The aims of this review are to recall the important results achieved with the use of HER-2 inhibitors in both gastric and colorectal cancer, and to discuss on the updates available on the role of HER-2 as prognostic and predictive factor in these malignancies.

## INTRODUCTION

HER-2 (ErbB-2, c-erbB2 or Her2/neu), a member of the HER-family that also includes HER-1 (Epidermal Growth Factor Receptor-EGFR, or ErbB1), HER-3 (ErbB3) and HER-4 (ErbB4), is a proto-oncogene that encodes for a 185-kDa plasma membrane-bound tyrosine kinase receptor, located on the chromosome 17 at q21 [1]. Its stimulation by extracellular signals leads to the activation of downstream pathways such as mitogen-activated protein kinase (MAPK), phosphoinositide-3-kinase (PI3K), phospholipase C and protein kinase C, inducing signal transduction and transcription [2, 3]. As such, HER-2 gene amplification and protein overexpression are involved in the pathogenesis and progression of several human cancers, thus they are often considered as a poor prognostic factor [4-6]. HER-2/neu overexpression has been reported in many epithelial malignancies including lung, prostate, bladder, pancreatic cancer and osteosarcoma. But it is mainly due to the revolutionary impact of anti-HER-2 therapy in breast cancer patients that the role of HER-2 and its blockade has been evaluated also in other tumor types, including gastric and colorectal cancers (CRC). Although several targeted agents have been tested in randomized trials [7, 8], only trastuzumab in HER2-positive patients [9] and ramucirumab in an unselected population [10, 11] are currently approved in gastric cancer. Similarly to gastric cancer, also CRC is a highly heterogeneous disease [12-14]. In such novel and more complex scenario, a better definition of HER-2 function in CRC has been achieved suggesting its involvement in disease pathogenesis as well as in the emergence of resistance to target therapy [15, 16]: as a result, HER-2 has been regarded as a potential therapeutic target.

**Figure 1:**
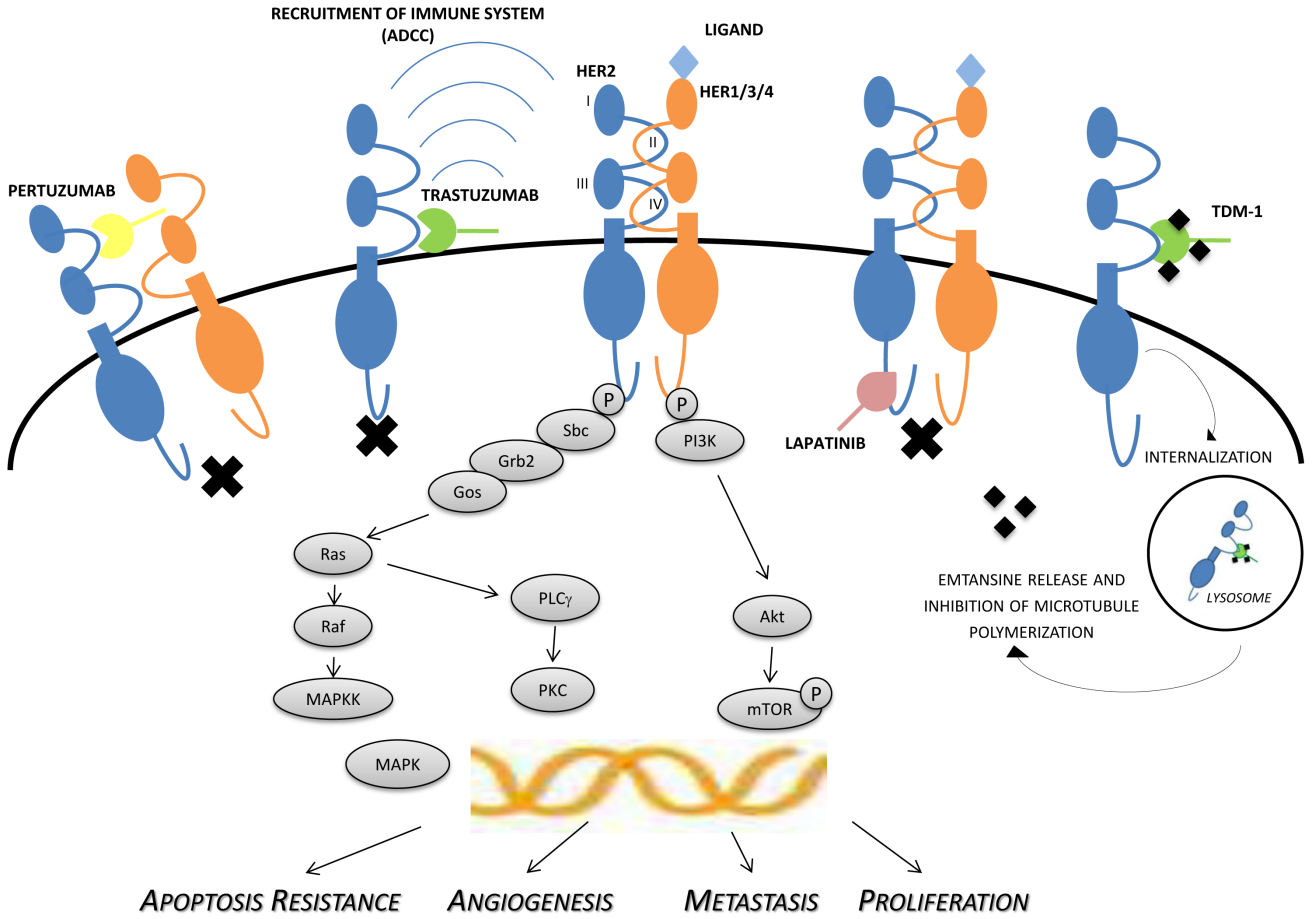
Mechanisms of actions of HER-2 inhibitors The figure shows the complex crosstalk between members of the HER-family and intracellular signaling, involved in proliferation, growth, invasion/metastases and angiogenesis. Activation of receptor kinase function occurs predominantly via ligand-mediated hetero- or homo-dimerization. In the case of HER-2, activation is also thought to occur in a ligand-independent manner, particularly when the receptor is found to be mutated or overexpressed. Trastuzumab is directed against the extracellular domain (IV) of HER-2, while pertuzumab binds to the extracellular dimerization domain (subdomain II). Immunologic mechanisms may also be involved in antitumor activity of trastuzumab, pertuzumab, and TDM-1, including ADCC. The potent small-molecule lapatinib inhibits the tyrosine kinases associated with HER-1 and HER-2, resulting in inhibition of phosphorylation and downstream signaling. ADCC: antibody-dependent cell-mediated cytotoxicity, HER: human epidermal growth factor receptor, MAPK: mitogen-activated protein kinase, MAPKK: mitogen-activated protein kinase kinase, mTOR: mammalian target of rapamycin, P: phosphate group, PI3K: phosphatidylinositol 3-kinase, PKC: protein kinase C, PLCγ: phospholipase C-gamma, TDM-1: trastuzumab emtansine.

The aims of this review are to recall the important results achieved with the use of HER-2 inhibitors in gastric and gastroesophageal junction cancers (as illustrated in Figure 1), and to discuss the updates on the role of HER-2 as prognostic and predictive factor in gastric and colorectal carcinomas.

## HER-2 AS A KEY TARGET IN GASTRIC CANCERS AND GASTROESOPHAGEAL JUNCTION CARCINOMAS

Gastric cancer is a worldwide health problem, causing approximately one million deaths per year and ranking as the third leading cause of cancer-related deaths [17, 18]. Although the role of HER-2 in this tumor has already been extensively investigated [19], and found to be frequently associated with invasion, high grade, and unfavorable prognosis, its overall prognostic role remains controversial [20-24] and appears to be stage-dependent.

HER-2 positivity in gastric cancer is usually established with immunohistochemistry (IHC) and further confirmed with in situ hybridization (ISH) methods. Currently, tumor scoring slightly diverges between small endoscopic biopsies and surgical specimens (see Table 1 for details). ISH analysis, either with fluorescence in situ hybridization (FISH) or chromogenic in situ hybridization (CISH), is required for tumors with an IHC score 2+ for which further assessments are needed to define the HER-2 status [25, 26]. Of note, HER-2 positivity may vary depending on the primary tumor location as well as the histology of gastric cancer. As a matter of fact, HER-2 overexpression/amplification is detected in more than 30% of the tumors arising from the gastroesophageal junction whereas less than 20% of the tumors arising from the gastric body are HER2-positive. Likewise, intestinal histology and diffuse histotype display a rate of HER-2 positivity of 34% and 6%, respectively [27]. The Cancer Genome Atlas project has identified four subtypes of gastric cancer based on their different molecular biology, which could actually explain such variability in HER-2 expression [28].

**Table 1:** Currently used HER-2 immunohistochemistry scoring system in gastric cancer depends on the available specimen

SCORE TO REPORT	HER-2 RECEPTOR OVEREXPRESSION ASSESSMENT	SCORING PATTERN: SURGICAL SPECIMEN	SCORING PATTERN: SMALL ENDOSCOPIC BIOPSIES (AT LEAST 6-8 DIAGNOSTIC FRAGMENTS)
**0**	Negative	No reactivity or membranous reactivity in <10% of tumor cells	No reactivity in any tumor cell
**1+**	Negative	Faint/barely perceptible incomplete membranous reactivity in ≥10% of tumor cells	Tumor cell cluster with a faint/barely perceptible incomplete membranous reactivity irrespective of percentage of tumor cells stained
**2+**	Equivocal	Weak to moderate complete, basolateral or lateral membranous reactivity in ≥10% of tumor cells	Tumor cell cluster with a weak to moderate complete, basolateral or lateral membranous reactivity irrespective of percentage of tumor cells stained
**3+**	Positive	Strong complete, basolateral or lateral membranous reactivity in ≥10% of tumorcells	Tumor cell cluster with a strong complete, basolateral or lateral membranous reactivity irrespective of percentage of tumor cells stained

HER-2: human epidermal growth factor receptor 2.

The relevant biological role played by HER-2 eventually led to explore its potential as a therapeutic target. ToGA, a large international randomized phase III study, investigated the efficacy and safety of adding trastuzumab to combination chemotherapy of a fluoropyrimidine (capecitabine or 5-FU) plus cisplatin for the upfront treatment of HER2-positive advanced gastric cancer patients [9]. The study enrolled 594 patients in 24 countries. This practice-changing study showed that the addition of trastuzumab to backbone chemotherapy significantly prolonged the median overall survival (OS) compared with chemotherapy alone (13.8 months *vs*. 11.1 months, HR 0.74, 95%CI 0.60-0.91); of note, a post-hoc analysis showed that the overall survival gain was even greater (+4.2 months, HR 0.65) for patients with higher HER-2 expression, i.e., IHC HER-2 3+ score or IHC HER-2 2+ score and amplified FISH (16.0 months *vs*. 11.8 months, HR 0.65, 95%CI 0.51-0.83) [9]. Full screening results from the ToGA study have been recently reported, including data on IHC and FISH testing. A total of 3,803 patients were screened for HER-2 status; either IHC or FISH testing was successful in 3,665 patients. Concordance between the two methods was 87.2%; HER-2 overexpression/amplification rates were similar for European and Asian patients, but slightly lower for American patients. As previously reported, HER-2 overexpression or amplification was more common in patients with intestinal histology compared with those with diffuse histology (31.8 % *vs*. 6.1%) and in gastroesophageal junction cancer than in gastric cancer (32.2% *vs*. 21.4%), suggesting a different etiology and pathogenesis between proximal and distal gastric cancers [29]. However, a critical factor for the selection of potential responders to a trastuzumab-based therapy remains the intratumoral heterogeneity of HER-2 amplification [30, 31] which, together with a lack of uniformity and reproducibility of the criteria used for defining HER-2 positivity, could be responsible for the variable incidence rates reported in literature [32]. Following the positive results of ToGA trial, further investigations have been conducted in order to: a) define the optimal dose of trastuzumab in combination with chemotherapy; b) extend the addition of trastuzumab to other disease settings (i.e. preoperative or perioperative) and c) test the efficacy of novel HER-2 inhibitors either alone or in combination with trastuzumab.

The identification of the most appropriate dose of trastuzumab in addition to chemotherapy is currently being pursued within the HELOISE trial, a randomized phase III study, which compares two different dosing regimens (loading dose of 8 mg/kg followed by either 6 mg/kg or 10 mg/kg of trastuzumab given every 3 weeks) in association with cisplatin/capecitabine chemotherapy, as first-line therapy in patients with HER2-positive metastatic gastric or gastro-esophageal junction adenocarcinoma. Primary study endpoints are safety and tolerability of an alternative trastuzumab dosing regimen based on a post-hoc analysis of ToGA trial [33]. In this trial, eligible patients have poor prognostic factors as ECOG performance status 2, primary tumor in site and metastatic involvement of at least two organs. In ToGA trial, trastuzumab was administered with an initial loading dose of 8 mg/kg followed by a maintenance dose of 6 mg/kg every 3 weeks. A total of 1,419 serum concentrations from 266 patients had a non-linear two-compartment pharmacokinetics (PK) with parallel linear and non-linear elimination. The non-linear elimination pathway of trastuzumab is believed to represent a target-mediated clearance process associated with the binding to the extracellular domain of the HER-2 protein. For the dosing regimen investigated in the study, the non-linear pathway was not saturated continuously, leading to a change in the overall trastuzumab clearance between consecutive infusions. Based on the predicted concentrations, the total clearance was stable within the first 3 days but increases by 48% as concentrations decline. A post-hoc Kaplan Meier survival analysis was conducted with patients stratified according to quartile of trastuzumab trough concentration in treatment at cycle 1. A significant difference in survival was observed according to quartile of trough trastuzumab concentrations in cycle 1 (p < 0.0001). Median survival time for patients within the lowest quartile of concentrations was 7.7 months (95% CI 6.3-10.6 months), which was 7 to 10 months shorter than median survival time reported for patients with trough concentrations within other quartiles [34]. Whether the addition of trastuzumab is beneficial also in settings other than metastatic has also been investigated in a number of studies which, as a matter of fact, have already shown favorable outcomes of trastuzumab-containing perioperative chemotherapy. In the explorative HERFLOT phase II study, the activity of trastuzumab in addition to perioperative FLOT (5-FU, leucovorin, oxaliplatin and docetaxel) is being evaluated. The primary study endpoint is the rate of pathological complete response (pCR) evaluated by a central reference pathologist. Preliminary data presented on an interim analysis revealed no unexpected safety issues and a pCR achieved in more than 20% patients. Final results are awaited later this year [35]. Similarly, the PETRARCA phase II/III trial of the AIO Group is evaluating the addition of trastuzumab and pertuzumab to FLOT *vs* FLOT alone in perioperative setting [36]. Moreover, the INNOVATION phase II study is assessing the activity of trastuzumab or trastuzumab plus pertuzumab in addition to perioperative standard chemotherapy [37]. In the TOXAG phase II trial, safety and tolerability of oxaliplatin, capecitabine and trastuzumab combination and radiotherapy as adjuvant therapy in surgically resected HER2-positive gastric or gastroesophageal junction cancer are being evaluated [38]. Finally, RTOG 1010 phase III is enrolling patients with esophageal HER2-positive adenocarcinoma in order to establish the efficacy of adding trastuzumab to chemoradiation [39].

Meanwhile, novel HER-2 inhibitors are also being studied in gastric cancer: lapatinib ditosylate, a dual anti-EGFR and anti-HER-2 tyrosine kinase inhibitor, was firstly tested in the TRIO-013/LOGiC trial designed to evaluate the efficacy and safety of the oral inhibitor in combination with capecitabine and oxaliplatin as first-line treatment of advanced or metastatic HER2-positive esophagogastric cancer. In this study, 545 patients were randomized 1:1 to receive CAPOX q21 (oxaliplatin 130 mg/sqm, day 1, capecitabine 850 mg/sqm/bid days 1-14) plus lapatinib (1,250 mg daily given continuously) or placebo. The primary endpoint was not met with an HR for OS of 0.91 (95% CI 0.73-1.12; *p* = 0.35), and a median OS of 12.2 months for standard treatment *vs*. 10.5 months in the experimental arm. Median progression-free survival (PFS) was 6.0 months (95% CI, 5.6 to 7.0) in experimental arm and 5.4 months (95% CI, 4.4 to 5.7) in control arm with an HR of 0.82 (95% CI,0.68-1.00; *p* = 0.0381). Overall Response Rate (ORR) was 53% (95% CI, 46.4-58.8) in the experimental arm and 39% (95% CI, 32.9-45.3, *p* = 0.0031) in the placebo arm [40]. In the open-label, phase III TyTAN trial, 261 HER2-positive advanced gastric cancer Asian patients were randomized to receive weekly paclitaxel with or without lapatinib, as second-line treatment. Once again, no benefit was demonstrated in terms of OS (HR 0.84, 95% CI 0.64-1.11; *p* = 0.10) or PFS (HR 0.85, 95%CI 0.63-1.13; *p* = 0.24) for the use of lapatinib, though a statistically significant improvement in RR was noted (27% v 9%, OR 3.85, 95%CI 1.80-8.87; *p* = 0.001) [41].

Possible reasons for these negative results are the high proportion of patients with IHC HER-2 1+score, which might have diluted the benefit of lapatinib in patients with IHC HER-2 3+ score, as well as the different rate of potentially active third-line treatments between study arms. Moreover, in TRIO-013/LOGiC trial, no correlation was observed between IHC status and OS benefit. Despite these uncertainties, lapatinib was not approved for gastric cancer. Key results of the most important randomized trials are summarized in Table 2. Ongoing studies might better define its role in combination with other targeted agents. Pertuzumab, a recombinant, humanized immunoglobulin IgG1κ monoclonal antibody which binds to the extracellular domain of HER-2 preventing its heterodimerization with other members of the HER-family (HER-1, HER-3, and HER-4), has been studied to overcome trastuzumab resistance. Pertuzumab and trastuzumab bind to distinct epitopes of HER-2 without competing with each other and have complementary mechanisms of HER-2 signaling disruption, resulting in synergistic antiproliferative activity both *in vitro* and *in vivo*. Based on the promising results from preclinical trials [42], the encouraging activity reported in early clinical trials enrolling gastric cancer patients [43], and the great efficacy of the combination of trastuzumab and pertuzumab in breast cancer [44], the phase III JACOB trial (BO 25114) was designed to assess whether the addition of pertuzumab to cisplatin, 5-Fluorouracil and trastuzumab may further improve the survival of HER2- positive gastric cancer patients with an acceptable safety profile [45]. Investigators reached the recruitment target of 780 patients enrolled in the JACOB study on January 2016 and results from this important trial are eagerly awaited. Moreover, an ongoing randomized phase IIa study is enrolling previously untreated advanced gastric cancer patients to better characterize the pharmacokinetics and safety of pertuzumab [43]. Novel strategies to target HER-2 include the combination of cytotoxic agents and antibodies with high activity like trastuzumab emtansine (TDM-1). This antibody, specifically designed to deliver an antiproliferative agent to HER-2 overexpressing cancer cells, conjugates the targeted anti-HER-2 activity of trastuzumab with the intracellular cytotoxic effect of emtansine, a tubulin polymerase inhibitor [46]. In a multicenter, randomized Phase II/III study the efficacy and safety of TDM-1 was compared to standard taxane treatment in patients with HER2-positive pretreated advanced gastric cancer. Participants were randomized to receive either trastuzumab emtansine 3.6 mg/kg every 3 weeks or trastuzumab emtansine 2.4 mg/kg every week or single-agent taxane (docetaxel or paclitaxel as per investigator choice). The aim of the first phase of the study was to choose the optimal TDM-1 dose and schedule. Despite better safety profile of TDM-1, accrual has been prematurely discontinued for failure of primary endpoint at first interim analysis [47]. Although a number of molecular mechanisms underpinning resistance to trastuzumab-based therapies have been already identified, a thorough investigation of such mechanisms will help clinicians to appropriately design future trials [8, 48].

**Table 2:** Principal results with HER-2 inhibitors in advanced HER2-positive gastric cancer

TRIAL	REGIMEN		PHASE	LINE	PRIMARY ENDPOINT	ORR (%)	PFS (months)	OS (months)
	**Experimental Arm**	**Control Arm**						
ToGA NCT01041404	Capecitabine or iv 5-FU + Cisplatin + Trastuzumab	Capecitabine or iv 5-FU + Cisplatin	III	I	OS	47.3 vs. 34.5 OR = 1.70, 95% CI 1.22 – 2.38, *p* = 0.0017	6.7 *vs*. 5.5 HR = 0.71, 95% CI 0.59-0.85, *p* = 0.0002	13.8 *vs*. 11.1 HR = 0.74, 95% CI 0.60-0.91, *p* = 0.0046
LOGiC NCT00680901	Capecitabine + Oxaliplatin + Lapatinib	Capecitabine + Oxaliplatin + Placebo	III	I	OS	53 (95% CI 46.6-59.3) vs. 40 (95% CI 33.6-46.4)	6.0 *vs*. 5.4 HR = 0.86, 95% CI 0.71-1.04, *p* = 0.10	12.2 *vs*. 10.5 HR = 0.91, 95% CI 0.73-1.12, *p* = 0.3492
TyTAN NCT00486954	Paclitaxel + Lapatinib	Paclitaxel + Placebo	III	II	OS	27 vs. 9 OR = 3.85, 95% CI 1.80-8.87, *p* < 0.001	5.4 *vs*. 4.4 HR = 0.85, 95% CI 0.63-1.13, *p* = 0.2441	11.0 *vs*. 8.9 HR = 0.84, 95% CI 0.64-1.11, *p* = 0.2088
GATSBY NCT01641939	TDM-1 3.6 mg/kg q21 or TDM-1 2.4 mg/kg q7	Paclitaxel or Docetaxel	II/III	II	OS	20.6 vs 19.6	2.7 *vs* 2.9 HR=1.13, 95% CI 0.89-1.43, *p* = 0.31	7.9 *vs* 8.6 HR= 1.15, 95% CI 0.87-1.51, *p* = 0.86

5-FU: 5-fluorouracil, CI: confidence interval, HR: hazard ratio, OR: odds ratio, iv: intravenous, ORR: overall response rate, OS: overall survival, PFS: progression-free survival, TDM-1: trastuzumab emtansine.

One of the possible mechanisms associated with trastuzumab resistance is the deregulation of HER-2 downstream signal, including the PI3K/AKT/mTOR pathway. It is well known that PIK3CA mutations and PTEN inactivation result in constitutive activation of the downstream signals [49]. Everolimus, an orally administered mTOR inhibitor, showed enhanced 5-FU-induced apoptosis in gastric cancer cells with HER-2 amplification and promising activity in preclinical and early clinical trials [50, 51]: such results, however, have not been confirmed in the phase III GRANITE-1 trial. [52]. It is uncertain whether the combination of HER2-targeted agents and mTOR inhibitors might provide benefit in patients with HER2-positive gastric cancer who became resistant: the identification of predictive biomarkers remains crucial for optimizing efficacy.

Afatinib, an oral irreversible inhibitor of tyrosine kinase receptors targeting HER-1, HER-2 and HER-4, may help overcoming trastuzumab resistance. Preliminary data of a phase II study enrolling patients with metastatic HER2-positive (IHC 3+ or FISH >2.0) esophagogastric cancer with disease progression whilst on a trastuzumab-containing regimen have been recently presented [53, 54] with demonstration of a 40% disease control.

As pan-HER inhibitors have shown significant antitumor effects *in vitro* as well as in xenograft model of HER2-positive gastric cancer [55], the efficacy and safety of dacomitinib, an irreversible pan-HER tyrosine kinase inhibitor, was investigated in advanced gastric cancer patients. Twenty-seven pretreated gastric cancer patients received dacomitinib 45 mg once daily continuously every 4 weeks. The 4-months PFS rate, the primary endpoint of the study, was 22.2% and median PFS was 2.1 months (95% CI 2.3-3.4). Disease control rate was 40.7% (95% CI 21.9-59.6%). Median OS was 7.1 months (95% CI 4.4-9.8). The most common adverse events were skin rash, diarrhea, and fatigue, while no treatment-related deaths were seen [56].

Moreover, data suggest that HER-3 might play a key role in tumorigenesis and act as a mediator of resistance to HER-2 inhibitors. MM-111, a novel molecule that inhibits heregulin-activated HER-3 signaling, has been shown to mitigate HER3-mediated resistance in preclinical gastric models [57]; thus, it is being tested within different trials in order to assess whether its addition might lead to an increased antitumor activity of trastuzumab and paclitaxel. [58]. Recent advances in HER-2 biology have expanded the use of HER-2 inhibitors in gastric cancer patients. Nevertheless, the underpinning molecular mechanisms of gastric cancer progression as well as the strategies to overcome resistance need to be further elucidated. International collaborative efforts to improve the knowledge on the background biology of this disease are ongoing (Table 3 and Table 4), as well as clinical and translational studies testing other novel HER-2 inhibitors.

**Table 3:** Principal ongoing trials in neoadjuvant HER2-positive gastric or gastroesophageal cancer

TRIAL	REGIMEN		PHASE	PRIMARY ENDPOINT
	Experimental Arm	Control Arm		
HERFLOT NCT01472029	5-FU + Leucovorin + Oxaliplatin + Docetaxel + Trastuzumab 6 mg/kg iv loading dose, followed by 4 mg/kg iv every 2 weeks	5-FU + Leucovorin + Oxaliplatin + Docetaxel	II	pCR
PETRARCA NCT02581462	5-FU + Leucovorin + Oxaliplatin + Docetaxel + Trastuzumab 8 mg/kg iv loading dose, followed by 6 mg/kg iv every 3 weeks +Pertuzumab 840 mg every 3 weeks	5-FU + Leucovorin + Oxaliplatin + Docetaxel	II/III	pCR/PFS
INNOVATION NCT02205047	5-FU/Capecitabine + Cisplatin + Trastuzumab/Pertuzumab	5-FU/Capecitabine + Cisplatin	II	pCR
TOXAG NCT01748773	RT at Total dose of 45 Gy divided into 25 doses +Capecitabine + Oxaliplatin + Trastuzumab 8 mg/kg iv loading dose, followed by 6 mg/kg iv every 3 weeks	//	II	Safety
RTOG 1010 NCT01196390	RT + Paclitaxel + Carboplatin + Trastuzumab	RT + Paclitaxel + Carboplatin	III	DFS

5-FU: 5-fluorouracil, OS: overall survival, PFS: progression-free survival, RT: radiotherapy, iv: intravenous, pCR: pathological complete response, DFS: disease free survival.

**Table 4:** Principal ongoing trials in advanced HER2-positive gastric cancer

TRIAL	REGIMEN		PHASE	LINE	PRIMARY ENDPOINT
	Experimental Arm	Control Arm			
HELOISE NCT01450696	Capecitabine + Cisplatin + Trastuzumab 8 mg/kg iv loading dose, followed 10 mg/kg iv every 3 weeks	Capecitabine + Cisplatin + Trastuzumab 8 mg/kg iv loading dose, followed by 6 mg/kg iv every 3 weeks	III	I	OS
JACOB NCT01774786	Capecitabine/5-FU + Cisplatin + Trastuzumab + Pertuzumab	Capecitabine/5-FU + Cisplatin + Trastuzumab + Placebo	III	I	OS
NCT01774851	Paclitaxel + Trastuzumab + MM-111	Paclitaxel + Trastuzumab	II	II	PFS

5-FU: 5-fluorouracil, OS: overall survival, PFS: progression-free survival. iv: intravenous

## HER-2: A NOVEL POTENTIAL TARGET IN COLORECTAL CANCER

In Western countries, CRC is one of the most common malignancies being the second leading cause of cancer-related death in the United States [17]. Despite the treatment advances achieved so far, with OS overcoming 30 months in genetically selected patients, metastatic CRC remains an incurable disease [59-62]. It is worth noting that, although most of the patients who have exhausted all standard treatments usually have a limited survival [63, 64], some of them may still have a good performance status and strongly desire further treatment. Paramount discoveries about the molecular biology underpinning CRC have been made [65], but we are still far from a thorough understanding of the tumor biology and the role of HER-2 overexpression and amplification in CRC.

HER-2 protein is usually weakly expressed in the membrane and/or cytoplasm of colonic epithelium, whereas the membrane of tumor cells may have various staining patterns [66].

The incidence of HER-2 expression in colon adenocarcinoma ranges from 0% to 83% [67-69] and reports on HER-2 gene amplification and HER-2 overexpression in metastatic CRC have been discordant. It is commonly agreed that such wide range may reflect differences in technical approaches, antibodies used, and scoring protocols, as well as study biases associated with patient selection [70, 71]. Most of the studies showed membranous overexpression rates between 0 and 15%. Of note, some studies reported higher rates of both membranous and cytoplasmic overexpression, up to 60%. Major hurdles in comparing HER-2 IHC studies in CRC are the variety of antibodies used as well as how technical issues are differently handled across protocols such as tissue fixation, slide storage procedures, antigen retrieval, and incubation time. To overcome such problems, a standard and reproducible staining procedure is highly needed. Overall, about 5% of CRC have HER-2 membranous overexpression, far lower than what it is observed in breast cancer [72, 73], while the cytoplasmic overexpression varies strongly with an average of 30%. However, it is likely that this value is underestimated due to the loss of HER-2 antigen in older tissue samples [74].

The disagreement in evaluating HER-2 expression at every level (nuclear, cytoplasmic and cell surface) emphasizes the limitations of using IHC evaluation alone and points out the need for further predictive biomarkers for HER2-targeted therapies in tumors displaying overexpression in gene copy number, mRNA and receptor protein [71].

Several studies analyzed HER-2 overexpression in CRC with genomic techniques such as fluorescence in situ hybridization (FISH), reverse transcription polymerase chain reaction (RT-PCR), Southern blotting, and Northern blotting. Altogether, these results confirmed that IHC 3+ overexpression is conclusive for gene amplification, whereas 2+ staining may be equivocal and likely associated with other mechanisms of genetic overexpression [74].

In a retrospective study, Tu et al. described a consistency between the levels of HER-2 protein expression determined by IHC and HER-2 gene amplification determined by FISH in 102 HER-2 overexpressed CRC samples. A relatively high level of consistency was observed between IHC 0/1+/3+ with FISH (24.5% of the IHC 3+ cases showed HER-2 gene amplification by FISH and none of the 20 randomly selected IHC 0/1+ cases demonstrated FISH amplification); however, there was a low level of consistency between IHC 2+ results and FISH. Of note, no association between HER-2 overexpression or gene amplification and survivorship was seen. [75]

In CRC, the clinical significance of HER-2 is still controversial. While some data seem to link HER-2 overexpression to decreased survival, suggesting that HER-2 may be a potential negative prognostic factor, other studies failed to confirm similar results [74]. Additionally, it is unclear whether HER-2 may be exploited as a potential therapeutic target in CRC patients [67].

CRC is a highly heterogeneous disease meaning, from a practical point of view, that some of the cellular clones will be sensitive to specific therapies while others will be not. Moreover, this heterogeneity dynamically varies under treatment pressure: while sensible clones start to disappear, resistant clones may eventually emerge and expand leading to disease progression. At this point, a new treatment may overcome resistance to previous compounds, determining another cancer clone remodeling [12-14]. As such, the selection of patients for a specific treatment is fundamental and, when available, predictive factors to both response and resistance are to be considered. Cetuximab and panitumumab, two epidermal growth factor receptor (EGFR) inhibitors approved for the treatment of metastatic CRC, are a stark example of this. KRAS, NRAS, BRAF, and possibly PIK3CA mutations prevent the efficacy of these drugs, confirming that constitutive activation of parallel or downstream pathways may bypass EGFR inhibition. Since EGFR may also heterodimerize with other members of HER family, it is reasonable to consider HER-2 as an additional response predictor [16].

In truth, HER-2 amplification seems to be associated with KRAS wild-type tumors and may cause acquired resistance to EGFR inhibition [15]. In the largest case series ever published, including tissue samples of 1,645 primary CRC patients, HER-2 status was assessed by IHC using the monoclonal antibody SP3. IHC 3+ score were reported as positive, 2+ score as equivocal, and 0/1+score as negative. Tumors scoring 2+ were further evaluated by CISH to assess HER-2 amplification. Only 9 cases were upfront scored as HER2-positive, while 35 cases showed an equivocal result. All cases scored 2+ and 3+, and 54 randomly selected 0/1+ cases were additionally tested on whole tissue sections. Overall, 1.6% of the whole CRC cohort was HER2-positive (26 out of 1,645). No differences between HER-2 status on primary tissue sample and resected locoregional nodes were reported. HER-2 positivity significantly correlated with higher stage at diagnosis and with the presence of nodal metastasis. Although not statistically significant, the rate of HER-2 positivity was higher in rectal than in colon cancers. Despite a trend toward worse prognosis, HER2-positivity had no significant impact on survival [76]. In line with these results, other recent series including a smaller number of patients have confirmed a similar low rate of HER-2 positivity in patients with CRC [66, 77] or rectal cancer [78].

In order to explore whether HER-2 was overexpressed and/or amplified in tumors from patients resistant to anti-EGFR antibodies, Bertotti et al. used direct transfer xenografts as a new strategy to anticipate clinical findings and to possibly optimize tailored treatment for these patients. For each tumor specimen, some fragments were subcutaneously implanted in immunodeficient mice and then expanded to generate a pair of independent xenograft lines for each patient (xenopatients). HER-2 amplification/overexpression was reported only in KRAS wild-type tumors that progressed on cetuximab or panitumumab [15], suggesting that HER-2 amplification may limit EGFR-inhibitors activity in RAS wild-type tumors.

Whether HER-2 gene copy number status may influence the response to cetuximab or panitumumab has also been retrospectively assessed in a large cohort of patients, who were divided into three groups according to their FISH evaluation. The results of this analysis showed that HER-2 amplification was associated with EGFR-inhibitors resistance and affected both PFS and OS [16]. Such findings support the results by Bertotti et al. and those by Yonesaka et al., who highlighted how the activation of ERBB2 signaling causes resistance to cetuximab leading to a worse prognosis [79].

The potential role of HER-2 in inducing resistance in CRC cells exposed to EGFR-inhibitors, was further investigated in a preclinical study where the impact of somatic mutations by introducing the HER2 V842I mutation, the most prevalent HER-2 mutation identified in CRC samples according to the Cancer Genome Atlas [80], into the cetuximab-sensitive colorectal cell line DiFi was assessed. The introduction of the V842I mutation caused a 40- to 100-fold shift in the half maximal inhibitory concentration (IC50) values of both cetuximab and panitumumab. Moreover, the impact of five HER-2 mutations in another cetuximab-sensitive CRC cell line (NCI-H508) was explored showing that all mutations led to the emergence of EGFR-inhibitors resistance. Western blots of the EGFR-HER2 signaling pathways suggested that the mechanism of anti-EGFR resistance consisted in sustaining MAPK phosphorylation. Based on preclinical studies, which showed that HER-2 mutations can be strongly inhibited by nanomolar doses of neratinib or afatinib [81, 82], both irreversible HER2/EGFR tyrosine kinase inhibitors were tested on DiFi and NCI-H508 cells transduced with HER-2 mutations leading to growth inhibition. Moreover, the authors sequenced the HER-2 gene of 48 CRC patient-derived xenografts (PDX) samples that were cetuximab-resistant and wild-type (WT) for KRAS, NRAS, BRAF, and PIK3CA (quadruple WT): four of these PDXs had HER-2 mutations. The effect of HER2-targeted therapies on two of these PDXs was assessed: single agent HER2-targeted therapy (with either trastuzumab, neratinib, or lapatinib) delayed the growth of these PDXs, while dual HER2-targeted therapy (with either trastuzumab plus neratinib or trastuzumab plus lapatinib) produced durable tumor regression [83].

Leto and coll. investigated the underlying cooperative mechanisms of co-targeting HER-2 with a monoclonal antibody and a small molecule. They evaluated the phosphorylation/activation of HER-2, HER-3, EGFR (HERs) and downstream transducers in HER2-overexpressing colorectal and gastric cancer cell lines; in addition, the *in vivo* outcome of antibody-mediated HER-2 blockade with trastuzumab, reversible HER2-inhibition by lapatinib, and irreversible HER2-inhibition by afatinib inpatient-derived tumorgrafts and cell-line xenografts was also assessed. The results of the study showed that trastuzumab monotherapy reduced HER-3 phosphorylation, with minor consequences on downstream transducers, while lapatinib acutely inhibited all HER receptors and effectors but led to delayed re-phosphorylation of HER-3 and EGFR and partial restoration of ERK and AKT activity. When combined with lapatinib, trastuzumab prevented HER3/EGFR reactivation and caused prolonged inhibition of ERK/AKT. Interestingly, afatinib alone was also very effective in counteracting the reinstatement of HER-3, EGFR, and downstream signaling activation. *In vivo*, the combination of trastuzumab and lapatinib or single-agent afatinib resulted in evident tumor shrinkage. Taken together, these findings showed that only prolonged inhibition of HER-3 and EGFR, achievable by dual blockade with trastuzumab and lapatinib or irreversible HER-2 inhibition by afatinib, led to regression of HER2-amplified gastrointestinal carcinomas [84]. These preclinical findings should be tested in clinical trials in order to validate the potential role of HER-2 as a target [85].

Whether HER-2 amplification is associated with specific clinico-pathological characteristics of CRC has also been studied. In a recent report, no association between HER-2 overexpression and gender, age, tumor site, size, depth of invasion, lymph node metastases or distant metastases was observed. In addition, no significant survival differences between tumors with or without HER-2 amplification were noted [75]. In other case series, HER-2 overexpression was not shown to be more frequently associated with microsatellite instability (MSI) or infiltrative tumor border, invasion depth, lymph node metastasis, distant metastasis, and perineural invasion. Both HER-2 overexpression and HER-2 gene amplification, however, were associated with rectal tumor location [66]. Accordingly, Missiaglia et al. observed that distal carcinomas are more often HER-2 amplified compared to proximal ones [86]. Concordance between primary tumor and distant metastases of HER-2 positivity has also been investigated [67, 87]. As a matter of fact, Lee et al. assessed in both primary and distant lesion KRAS and HER-2 status of 94 consecutive CRC patients who underwent curative resection of the primary tumor and liver or lung metastases. HER-2 protein overexpression was assessed by IHC, with only 2.1% of cases overexpressed. HER-2 amplification was observed in 10.1% of combined primary tissue and metastases, suggesting high levels of concordance between primary disease and paired secondary lesions [67]. In a retrospective Italian study that enrolled 50 CRC patients neurosurgically resected for secondary brain lesions, the overall HER-2 positivity of brain metastases was 12%, and a concordance rate of 89% between primary tumor and distant lesion was reported [87]. In line with these results, a high concordance between the primary tumor and liver metastasis was also described in another study [88] thus suggesting that metastatic lesions may also be suitable for anti-HER-2 therapy.

The predictive role of HER-2 in CRC has been recently unveiled. Unlike cetuximab and panitumumab, treatment response to trastuzumab highly depends on HER-2 protein overexpression and gene amplification. Since approximately 5% of all CRC patients have membranous overexpression, HER-2 inhibitors were supposed to be effective only in a small subgroup of patients, who should be therefore selected based on of their HER-2 expression pattern. The definition of the role of anti-HER-2 treatment for metastatic CRC has not been straightforward with two pivotal clinical trials prematurely closed due to a slow accrual: the low incidence of HER-2 overexpression was to blame for such failure [89, 90]. Moreover, a phase II study failed to show sufficient activity for the combination of capecitabine and lapatinib in patients with refractory intestinal adenocarcinoma [91], while preclinical synergy was demonstrated for the oral HER-2 inhibitor combined with panobinostat, a histone deacetylase inhibitor [75, 92]. Whether HER-2 inhibition could restore sensitivity to EGFR inhibitors was also studied. A phase I/II trial has been conducted to assess the efficacy and safety of cetuximab in combination with pertuzumab in patients with cetuximab-resistant metastatic CRC. Despite an encouraging RR, the study was prematurely stopped due to severe overlapping toxicities of the two compounds, which included severe skin rash, mucositis and diarrhea [93].

The HERACLES trial (HER2 Amplification for Colo-rectaL Cancer Enhanced Stratification) tested the combination of trastuzumab plus lapatinib or pertuzumab in metastatic HER2-amplified CRC patients. A validation study was conducted in order to develop a validated ERBB2 scoring system for colorectal cancer with the aim of identifying ERBB2-positive patients suitable for enrollment in the HERACLES trial [94]. In both archival test cohort (*n* = 256) and in the validation cohort (*n* = 830) a 5% of KRAS wild-type pretreated CRC patients were found to be HER2-positive. In the phase II HERACLES trial, 23 heavily pretreated KRAS wild-type, HER2-positive patients (IHC 3+ or 2+ and FISH positive with HER-2-to-CEP17 ratio >2), refractory to all standard agents including fluoropyrimidines, oxaliplatin, irinotecan, bevacizumab, and EGFR-inhibitors, were treated with trastuzumab and lapatinib. The primary trial endpoint was RECIST-determined objective RR.

Overall, 913 patients were screened, 44 were found to be HER2-positive and 23 were enrolled (2.3% of those screened). The Italian investigators observed 8 responses out of 23 treated patients (ORR 32%) and the trial met its primary endpoint before the enrollment was completed, with a median time to progression of 5.5 months (95%CI 3.7-9.8) and some long-lasting responders. HERACLES, the first precision medicine trial with positive results in advanced CRC, proved that the combination of trastuzumab and lapatinib is clinically effective in HER2-positive heavily pretreated metastatic CRC patients. As such, this combination needs to be further investigated in earlier treatment lines [95].

Finally, HER-2 can be immunogenic and generate antibodies and activation of T lymphocytes, suggesting that it can also be a target for T cell-directed immunotherapy [71]., which are 2, the role of immunotherapy with T cells genetically modified to express HER-2 ; this might be an interesting option that could be considered for those metastatic CRC patients displaying cytoplasmic HER-2 overexpression [71].

Taken together, these pre-clinical and clinical data, pose HER-2 as an attractive object of interest, mainly from a therapeutic point of view, in the treatment of metastatic CRC. The assessment of HER-2 status may therefore be useful both as predictive factor of response to treatment with EGFR-inhibitors both as the main therapeutic target in a percentage, albeit small, of patients suffering from metastatic CRC. In this context, it would be useful to plan clinical trials testing the activity of anti-HER2 agents not only as drugs able to restore the sensitivity to EGFR-inhibitors, but also in a first line setting. For these reasons, despite HER2-positivity is observed more often in RAS wt disease, it might be useful to assess the HER-2 status both since the diagnosis of metastatic CRC and subsequently during biopsy of a metastatic site.

## CONCLUSIONS

Although palliative chemotherapy may improve both patient's outcome and quality of life, its overall benefit is still limited. However, with the introduction of novel targeted therapies, the median survival of gastrointestinal malignancies is slowly but constantly improving, but a thorough understanding of CRC molecular biology is required to develop new targeted compounds.

Significant benefits have been observed with trastuzumab in HER2-positive tumors but the journey to a full implementation of anti-HER-2 therapy has just begun. Ongoing clinical trials are aiming at a better patient selection mainly according to the tumor molecular profile. Evidence that HER-2 overexpression drives progression and resistance to therapy in a limited subset of gastrointestinal cancers, thus becoming a valid drug target in this setting, has represented a major step forward in tailoring cancer therapy. However, anti-HER-2 treatment, even when tested in a selected biomarker-positive population, provides only modest survival gains [9, 95], given the inevitable development of acquired resistance. Intratumour heterogeneity, supporting clonal evolution under treatment pressure, is the main culprit for the emergence of resistance [97]. Additional oncogenic and epigenetic events have also been reported to confer resistance to HER-2 treatment in gastrointestinal cancers, suggesting that combination inhibitory strategies should be pursued to effectively prevent or delay the development of resistance [92]. Moreover, understanding secondary features that contribute to resistance, may allow the development of rational combination approaches to improve the efficacy of anti-HER-2 treatment in gastrointestinal cancers. To accomplish this, “liquid biopsies” allow a dynamic monitoring of clonal evolution, providing a valid tool to maximize anti-HER-2 treatment for gastrointestinal cancers [12].

This will ultimately help clinicians to administer the right treatment to the right patient increasing survival, decreasing side effects and finally improving quality of life.
